# Supplementary material for: Upper Limb Strikes Reactive Forces in Mix Martial Art Athletes during Ground and Pound Tactics
Source: Int J Environ Res Public Health. 2020 Oct 24;17(21):7782. doi: 10.3390/ijerph17217782 (PMC7660618; doi:10.3390/ijerph17217782)
Supplement: Supplementary file 1 [file ijerph-17-07782-s001.zip › Supplementary_material_1.docx]

**SUPPLEMENTARY MATERIAL 3  *Results of one-sided z-test testing hypothesis that our Fpeak values come from normal distributions with means and standard deviations reported in literature for straight punch and palm strike against the alternative that the our Fpeak mean is greater. CI = confidence interval. Higher boundary of the confidence intervals is equal to infinity in one-sided test.***

|  | **Author** | **p** | **CI lower boundary** | **z statistic** |
| --- | --- | --- | --- | --- |
| Straight Punch | de Souza et al (2017) | > 0.01 | 2966 | 342.05 |
|  | Bolander et al (2009) | > 0.01 | 2960 | 298.30 |
|  | Neto et al (2009) | > 0.01 | 2953 | 184.85 |
|  | Walilko et al (2005) | 1.00 | 2909 | -11.91 |
|  | Rat Tong Iam et al (2017) | > 0.01 | 2950 | 125.57 |
|  | Loturco et al (2016) | > 0.01 | 2953 | 156.36 |
|  | Chadli et al (2014) | > 0.01 | 2963 | 360.43 |
|  | Busko et al (2016) | > 0.01 | 2933 | 57.70 |
|  | Bingul et al (2017) | > 0.01 | 2945 | 61.07 |
|  | Pierce et al (2006) | > 0.01 | 2920 | 58.07 |
| Palm Strike | Neto et al (2012) | > 0.01 | 4088 | 92.35 |
|  | Neto et al (2009) | > 0.01 | 4108 | 225.61 |
|  | Neto et al (2008) | > 0.01 | 4124 | 830.11 |
